# 4-Bromo­methyl-7,8-dimethyl­coumarin

**DOI:** 10.1107/S1600536810049135

**Published:** 2010-11-30

**Authors:** Ramakrishna Gowda, K.V. Arjuna Gowda, Mahantesha Basanagouda, Manohar V. Kulkarni

**Affiliations:** a Department of Physics, Govt. College for Women, Kolar 563 101, Karnataka, India; b Department of Physics, Govt. College for Women, Mandya 571 401, Karnataka, India; c Department of Chemistry, Karnatak University, Dharwad 580 003, Karnataka, India

## Abstract

In the title mol­ecule, C_12_H_11_BrO_2_, all non-H atoms with the exception of the Br atom are essentially coplanar (r.m.s. deviation = 0.018 Å). The C—Br bond is inclined by 80.17 (12)° to this plane. The crystal structure is stabilized by weak C—H⋯O hydrogen bonds.

## Related literature

For potential synthetic applications of the title compound, see: Cui *et al.* (2007 ▶); Zhao *et al.* (2008 ▶). For related structures, see: Gowda *et al.* (2009 ▶, 2010 ▶).
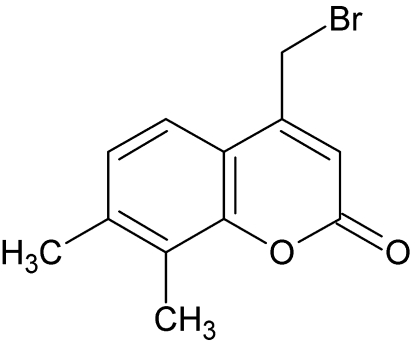


## Experimental

### 

#### Crystal data

C_12_H_11_BrO_2_
*M* *_r_* = 267.12
Monoclinic, 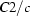

*a* = 18.5025 (14) Å
*b* = 9.8785 (7) Å
*c* = 13.1639 (10) Å
β = 118.908 (2)°
*V* = 2106.3 (3) Å^3^
*Z* = 8
Mo *K*α radiation
μ = 3.88 mm^−1^
*T* = 292 K
0.30 × 0.20 × 0.20 mm

#### Data collection

Bruker Kappa APEXII CCD diffractometer
Absorption correction: multi-scan (*SADABS*; Bruker 2004 ▶) *T*_min_ = 0.432, *T*_max_ = 0.571
14710 measured reflections
3610 independent reflections
2516 reflections with *I* > 2σ(*I*)
*R*_int_ = 0.026

#### Refinement

*R*[*F* ^2^ > 2σ(*F* ^2^)] = 0.041
*wR*(*F* ^2^) = 0.116
*S* = 1.05
3610 reflections
138 parameters
H-atom parameters constrained
Δρ_max_ = 1.78 e Å^−3^
Δρ_min_ = −0.73 e Å^−3^

### 

Data collection: *APEX2* (Bruker, 2004 ▶); cell refinement: *APEX2* and *SAINT* (Bruker, 2004 ▶); data reduction: *SAINT*; program(s) used to solve structure: *SIR92* (Altomare *et al.*, 1994) ▶; program(s) used to refine structure: *SHELXL97* (Sheldrick, 2008 ▶); molecular graphics: *ORTEP-3* (Farrugia, 1997 ▶) and *PLATON* (Spek, 2009 ▶); software used to prepare material for publication: *SHELXL97*.

## Supplementary Material

Crystal structure: contains datablocks global, I. DOI: 10.1107/S1600536810049135/lh5165sup1.cif

Structure factors: contains datablocks I. DOI: 10.1107/S1600536810049135/lh5165Isup2.hkl

Additional supplementary materials:  crystallographic information; 3D view; checkCIF report

## Figures and Tables

**Table 1:** Hydrogen-bond geometry (Å, °)

*D*—H⋯*A*	*D*—H	H⋯*A*	*D*⋯*A*	*D*—H⋯*A*
C12—H12*B*⋯O2^i^	0.97	2.40	3.342 (3)	163

Symmetry code: (i) 
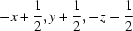
.

**Fig. 1.**
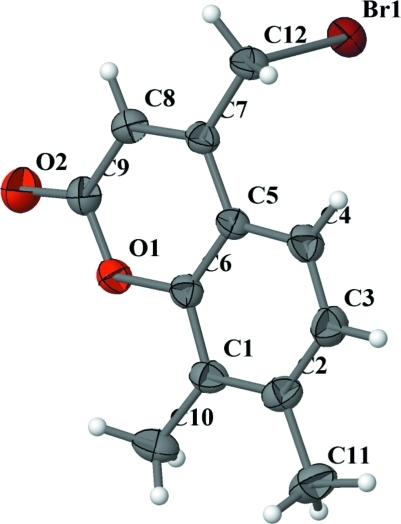
The molecular structure of the title compound shown with 50% probability displacement ellipsoids.

**Fig. 2.**
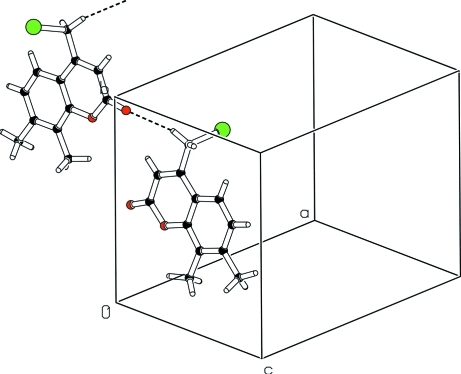
Part of the crystal structure showing weak C-H···O hydrogen bonds as dashed lines.

## supplementary crystallographic information

### Comment

The title compound has potential use in Heck and Suzuki cross coupling reactions (Cui *et al.*,  2007) and Negishi coupling reactions (Zhao *et al.*, 2008). In continuation of our work on the crystal structures of halogenated coumarin derivatives (Gowda *et al.*, 2009; 2010) herein we report crystal structure of title compound.

The molecular structure of the title compound is shown in Fig. 1. In the molecule, with the exception of the Br atom, all non-hydrogen atoms [C1-C12/O1/O2] are essentially planar [r.m.s. = 0.018Å]]. The C-Br bond is inclined [defined by the C7/C12/Br1 plane] by 80.17 (12)° to this plane. The crystal structure is stabilized by weak C-H···O hydrogen bonds (Fig. 2).

### Experimental

To a mixture of equimolar quantities of 2,3-dimethylphenol (0.1 mol) and 4-bromoethylacetoacetate (0.1 mol), sulfuric acid (30 ml) was added dropwise with stirring while maintaining the temperature between 273-278K. The reaction mixture was allowed to stand in ice chest overnight and the deep red coloured solution was poured into a stream of crushed ice. The solid which separated was filtered and washed with water and then with cold ethanol to yield a colourless compound which was recrystallized from acetic acid.

### Refinement

All H atoms were positioned geometrically and refined using a riding model with bond lengths 0.96 (methyl) or 0.93 Å (aromatic) and *U*_iso_(H) = 1.5*U*_eq_(C) for methyl groups and *U*_iso_(H) = 1.2*U*_eq_(C) for all other H atoms.

### Crystal data

**Table:**

C_12_H_11_BrO_2_	*F*(000) = 1072
*M**_r_* = 267.12	*D*_x_ = 1.685 Mg m^−^^3^
Monoclinic, *C*2/*c*	Mo *K*α radiation, λ = 0.71073 Å
Hall symbol: -C 2yc	Cell parameters from 5018 reflections
*a* = 18.5025 (14) Å	θ = 2.4–28.6°
*b* = 9.8785 (7) Å	µ = 3.88 mm^−^^1^
*c* = 13.1639 (10) Å	*T* = 292 K
β = 118.908 (2)°	Block, colourless
*V* = 2106.3 (3) Å^3^	0.30 × 0.20 × 0.20 mm
*Z* = 8	

### Data collection

**Table:**

Bruker Kappa APEXII CCD diffractometer	3610 independent reflections
Radiation source: fine-focus sealed tube	2516 reflections with *I* > 2σ(*I*)
graphite	*R*_int_ = 0.026
ω and φ scans	θ_max_ = 31.9°, θ_min_ = 2.4°
Absorption correction: multi-scan (*SADABS*; Bruker 2004)	*h* = −27→27
*T*_min_ = 0.432, *T*_max_ = 0.571	*k* = −14→14
14710 measured reflections	*l* = −19→13

### Refinement

**Table:**

Refinement on *F*^2^	Primary atom site location: structure-invariant direct methods
Least-squares matrix: full	Secondary atom site location: difference Fourier map
*R*[*F*^2^ > 2σ(*F*^2^)] = 0.041	Hydrogen site location: inferred from neighbouring sites
*wR*(*F*^2^) = 0.116	H-atom parameters constrained
*S* = 1.05	*w* = 1/[σ^2^(*F*_o_^2^) + (0.0574*P*)^2^ + 1.9612*P*] where *P* = (*F*_o_^2^ + 2*F*_c_^2^)/3
3610 reflections	(Δ/σ)_max_ = 0.001
138 parameters	Δρ_max_ = 1.78 e Å^−^^3^
0 restraints	Δρ_min_ = −0.73 e Å^−^^3^

### Special details

**Table:**

Geometry. All esds (except the esd in the dihedral angle between two l.s. planes) are estimated using the full covariance matrix. The cell esds are taken into account individually in the estimation of esds in distances, angles and torsion angles; correlations between esds in cell parameters are only used when they are defined by crystal symmetry. An approximate (isotropic) treatment of cell esds is used for estimating esds involving l.s. planes.
Refinement. Refinement of F^2^ against ALL reflections. The weighted R-factor wR and goodness of fit S are based on F^2^, conventional R-factors R are based on F, with F set to zero for negative F^2^. The threshold expression of F^2^ > 2sigma(F^2^) is used only for calculating R-factors(gt) etc. and is not relevant to the choice of reflections for refinement. R-factors based on F^2^ are statistically about twice as large as those based on F, and R- factors based on ALL data will be even larger.

### Fractional atomic coordinates and isotropic or equivalent isotropic displacement parameters (Å^2^)

**Table:**

	*x*	*y*	*z*	*U*_iso_*/*U*_eq_	
C1	0.36207 (15)	0.1296 (2)	0.0849 (2)	0.0332 (5)	
C2	0.39174 (15)	0.1512 (2)	0.2032 (2)	0.0363 (5)	
C3	0.40658 (16)	0.2836 (3)	0.24674 (19)	0.0398 (5)	
H3	0.4263	0.2974	0.3257	0.048*	
C4	0.39291 (16)	0.3930 (3)	0.17644 (19)	0.0366 (5)	
H4	0.4033	0.4797	0.2079	0.044*	
C5	0.36354 (14)	0.3756 (2)	0.05795 (17)	0.0289 (4)	
C6	0.34902 (13)	0.2433 (2)	0.01547 (17)	0.0292 (4)	
C7	0.34654 (14)	0.4850 (2)	−0.02371 (18)	0.0303 (4)	
C8	0.31617 (15)	0.4562 (2)	−0.13705 (19)	0.0354 (5)	
H8	0.3025	0.5272	−0.1896	0.043*	
C9	0.30411 (16)	0.3200 (2)	−0.1796 (2)	0.0371 (5)	
C10	0.34498 (19)	−0.0090 (2)	0.0328 (3)	0.0460 (6)	
H10A	0.3014	−0.0043	−0.0461	0.069*	
H10B	0.3287	−0.0674	0.0764	0.069*	
H10C	0.3939	−0.0443	0.0346	0.069*	
C11	0.4099 (2)	0.0349 (3)	0.2855 (3)	0.0530 (7)	
H11A	0.3594	−0.0110	0.2678	0.079*	
H11B	0.4350	0.0684	0.3637	0.079*	
H11C	0.4469	−0.0271	0.2778	0.079*	
C12	0.36044 (16)	0.6286 (2)	0.0163 (2)	0.0383 (5)	
H12A	0.3423	0.6421	0.0734	0.046*	
H12B	0.3287	0.6883	−0.0489	0.046*	
O1	0.32072 (11)	0.21805 (17)	−0.10098 (13)	0.0360 (4)	
O2	0.28162 (15)	0.2864 (2)	−0.27881 (15)	0.0554 (5)	
Br1	0.478174 (17)	0.67133 (3)	0.08473 (2)	0.04795 (12)	

### Atomic displacement parameters (Å^2^)

**Table:**

	*U*^11^	*U*^22^	*U*^33^	*U*^12^	*U*^13^	*U*^23^
C1	0.0327 (11)	0.0270 (10)	0.0415 (11)	0.0027 (9)	0.0190 (9)	0.0012 (9)
C2	0.0344 (12)	0.0387 (13)	0.0398 (11)	0.0035 (10)	0.0211 (10)	0.0077 (9)
C3	0.0438 (13)	0.0458 (13)	0.0298 (10)	−0.0008 (11)	0.0178 (9)	−0.0007 (9)
C4	0.0432 (13)	0.0338 (12)	0.0336 (10)	−0.0029 (10)	0.0192 (9)	−0.0068 (9)
C5	0.0311 (11)	0.0248 (9)	0.0302 (9)	0.0002 (8)	0.0143 (8)	−0.0018 (7)
C6	0.0300 (10)	0.0285 (10)	0.0293 (9)	0.0007 (8)	0.0144 (8)	−0.0022 (7)
C7	0.0296 (10)	0.0252 (10)	0.0348 (9)	0.0003 (8)	0.0145 (8)	−0.0006 (8)
C8	0.0386 (12)	0.0317 (11)	0.0333 (9)	−0.0003 (9)	0.0153 (9)	0.0036 (8)
C9	0.0383 (12)	0.0369 (12)	0.0323 (9)	−0.0018 (10)	0.0140 (9)	−0.0019 (9)
C10	0.0564 (16)	0.0264 (11)	0.0576 (14)	0.0007 (11)	0.0295 (13)	−0.0007 (10)
C11	0.0552 (17)	0.0542 (17)	0.0511 (14)	0.0062 (14)	0.0269 (13)	0.0211 (13)
C12	0.0402 (13)	0.0256 (10)	0.0434 (12)	0.0017 (10)	0.0158 (10)	−0.0003 (9)
O1	0.0464 (10)	0.0278 (8)	0.0312 (7)	−0.0013 (7)	0.0169 (7)	−0.0053 (6)
O2	0.0764 (15)	0.0524 (11)	0.0310 (8)	−0.0053 (11)	0.0208 (9)	−0.0057 (8)
Br1	0.04529 (18)	0.04007 (16)	0.04762 (16)	−0.00806 (11)	0.01383 (12)	0.00024 (10)

### Geometric parameters (Å, °)

**Table:**

C1—C6	1.393 (3)	C8—C9	1.433 (3)
C1—C2	1.394 (3)	C8—H8	0.9300
C1—C10	1.495 (3)	C9—O2	1.210 (3)
C2—C3	1.401 (4)	C9—O1	1.368 (3)
C2—C11	1.502 (3)	C10—H10A	0.9600
C3—C4	1.364 (4)	C10—H10B	0.9600
C3—H3	0.9300	C10—H10C	0.9600
C4—C5	1.392 (3)	C11—H11A	0.9600
C4—H4	0.9300	C11—H11B	0.9600
C5—C6	1.396 (3)	C11—H11C	0.9600
C5—C7	1.447 (3)	C12—Br1	1.959 (3)
C6—O1	1.382 (2)	C12—H12A	0.9700
C7—C8	1.346 (3)	C12—H12B	0.9700
C7—C12	1.492 (3)		
			
C6—C1—C2	117.3 (2)	C9—C8—H8	118.9
C6—C1—C10	120.4 (2)	O2—C9—O1	116.7 (2)
C2—C1—C10	122.3 (2)	O2—C9—C8	125.9 (2)
C1—C2—C3	119.6 (2)	O1—C9—C8	117.38 (19)
C1—C2—C11	121.2 (2)	C1—C10—H10A	109.5
C3—C2—C11	119.2 (2)	C1—C10—H10B	109.5
C4—C3—C2	121.7 (2)	H10A—C10—H10B	109.5
C4—C3—H3	119.1	C1—C10—H10C	109.5
C2—C3—H3	119.1	H10A—C10—H10C	109.5
C3—C4—C5	120.4 (2)	H10B—C10—H10C	109.5
C3—C4—H4	119.8	C2—C11—H11A	109.5
C5—C4—H4	119.8	C2—C11—H11B	109.5
C4—C5—C6	117.4 (2)	H11A—C11—H11B	109.5
C4—C5—C7	124.5 (2)	C2—C11—H11C	109.5
C6—C5—C7	118.09 (18)	H11A—C11—H11C	109.5
O1—C6—C1	115.77 (19)	H11B—C11—H11C	109.5
O1—C6—C5	120.65 (19)	C7—C12—Br1	109.33 (17)
C1—C6—C5	123.6 (2)	C7—C12—H12A	109.8
C8—C7—C5	119.4 (2)	Br1—C12—H12A	109.8
C8—C7—C12	120.0 (2)	C7—C12—H12B	109.8
C5—C7—C12	120.64 (19)	Br1—C12—H12B	109.8
C7—C8—C9	122.3 (2)	H12A—C12—H12B	108.3
C7—C8—H8	118.9	C9—O1—C6	122.16 (18)
			
C6—C1—C2—C3	0.3 (4)	C7—C5—C6—C1	−179.3 (2)
C10—C1—C2—C3	180.0 (2)	C4—C5—C7—C8	−178.3 (2)
C6—C1—C2—C11	−178.3 (2)	C6—C5—C7—C8	1.2 (3)
C10—C1—C2—C11	1.3 (4)	C4—C5—C7—C12	0.3 (4)
C1—C2—C3—C4	−0.1 (4)	C6—C5—C7—C12	179.7 (2)
C11—C2—C3—C4	178.6 (3)	C5—C7—C8—C9	−3.5 (4)
C2—C3—C4—C5	−0.1 (4)	C12—C7—C8—C9	178.0 (2)
C3—C4—C5—C6	0.0 (4)	C7—C8—C9—O2	−175.6 (3)
C3—C4—C5—C7	179.5 (2)	C7—C8—C9—O1	3.3 (4)
C2—C1—C6—O1	179.1 (2)	C8—C7—C12—Br1	−101.7 (2)
C10—C1—C6—O1	−0.5 (3)	C5—C7—C12—Br1	79.8 (2)
C2—C1—C6—C5	−0.4 (4)	O2—C9—O1—C6	178.2 (2)
C10—C1—C6—C5	180.0 (2)	C8—C9—O1—C6	−0.8 (4)
C4—C5—C6—O1	−179.3 (2)	C1—C6—O1—C9	179.1 (2)
C7—C5—C6—O1	1.2 (3)	C5—C6—O1—C9	−1.4 (3)
C4—C5—C6—C1	0.2 (3)		

### Hydrogen-bond geometry (Å, °)

**Table:**

*D*—H···*A*	*D*—H	H···*A*	*D*···*A*	*D*—H···*A*
C12—H12B···O2^i^	0.97	2.40	3.342 (3)	163

Symmetry codes: (i) −*x*+1/2, *y*+1/2, −*z*−1/2.
